# ‘Everything's fine, so why does it happen?’ A qualitative investigation of patients' perceptions of noncardiac chest pain

**DOI:** 10.1111/jocn.12841

**Published:** 2015-05-19

**Authors:** Rosie Webster, Andrew R Thompson, Paul Norman

**Affiliations:** ^1^ Department of PsychologyUniversity of SheffieldSheffieldUK; ^2^ Clinical Psychology UnitDepartment of PsychologyUniversity of SheffieldSheffieldUK

**Keywords:** accident and emergency nursing, anxiety, illness representations, medically unexplained symptoms, noncardiac chest pain, thematic analysis

## Abstract

**Aims and objectives:**

To examine patients' perceptions and experiences of noncardiac chest pain, within the framework of the common sense model.

**Background:**

Patients with noncardiac chest pain have good physical prognosis, but frequently suffer prolonged pain and psychological distress. The common sense model may provide a good framework for examining outcomes in patients with noncardiac chest pain.

**Design:**

Qualitative thematic analysis with semi‐structured interviews.

**Methods:**

In 2010, participants recruited from an emergency department (*N *= 7) with persistent noncardiac chest pain and distress were interviewed using a semi‐structured schedule, and data were analysed using thematic analysis.

**Results:**

Seven themes were identified; six of which mapped onto core dimensions of the common sense model (identity, cause, timeline, consequences, personal control, treatment control). Contrary to previous research on medically unexplained symptoms, most participants perceived psychological factors to play a causal role in their chest pain. Participants' perceptions largely mapped onto the common sense model, although there was a lack of coherence across dimensions, particularly with regard to cause.

**Conclusion:**

Patients with noncardiac chest pain lack understanding with regard to their condition and may be accepting of psychological causes of their pain.

**Relevance to clinical practice:**

Brief psychological interventions aimed at improving understanding of the causes of noncardiac chest pain and providing techniques for managing pain and stress may be useful for patients with noncardiac chest pain.

**What does this paper contribute to the wider global community?**

- Patients with NCCP may require further explanation regarding potential causes of their symptoms.
- Brief psychological intervention could be beneficial for patients with NCCP.

## Introduction

Noncardiac chest pain (NCCP) is a common condition, characterised by chest pain with no apparent serious or cardiac cause. Between 30–60% of the 700,000 patients attending emergency departments (EDs) for chest pain each year do not receive a cardiac diagnosis (Mayou & Thompson 2002, Goodacre *et al*. 2005, Eken *et al*. 2010). Guidelines recommend that staff simply explain the noncardiac nature of the pain to patients (National Institute for Health & Care Excellence 2010), despite evidence that providing reassurance that test results are negative is often insufficient to reduce patients' anxiety about cardiac problems (McDonald *et al*. 1996). Despite having excellent physical prognosis (Papanicolaou *et al*. 1986), patients with NCCP may experience elevated levels of anxiety, reduced quality of life (QoL), continued episodes of chest pain, and consequently high use of health care services (Goodacre *et al*. 2001, Webster *et al*. 2012). NCCP is therefore not only linked to ongoing patient distress but also places a burden on health care resources, in addition to indirect economical costs that may result through lost work days (Eslick *et al*. 2002).

### Background

Previous research on the predictors of psychological and physical outcomes in patients with NCCP has lacked a strong theoretical basis (Webster *et al*. 2012). The common sense model of illness representations (CSM, Leventhal *et al*. 1980) may provide a suitable theoretical model for examining the predictors of outcomes in patients with NCCP (Webster *et al*. 2012). The CSM proposes that when faced with a health threat or illness, such as the experience of chest pain, people form a representation of the health threat, through lay knowledge of the illness and input from others. Illness representations are based around the dimensions of the perceived causes of the illness, consequences of the illness, identity (i.e. the label given to the illness and the symptoms associated with it), expected timeline of the illness, cure/controllability of the illness (personal and treatment), one's emotional response to the illness and illness coherence (understanding). More negative illness representations (e.g. perceived worse consequences and longer timeline, less belief in the curability/controllability of the illness, less understanding) are hypothesised to be related to more negative physical and psychological outcomes.

The CSM has been applied to NCCP, with findings demonstrating that negative illness representations are related to psychological (Jonsbu *et al*. 2012) and physical (Schroeder *et al*. 2012) outcomes in patients with NCCP. Webster *et al*. (2014b) found that illness representations were related to elevated anxiety and depression and poorer QoL. Therefore, this model may be helpful in developing interventions for patients with NCCP and psychological morbidity. Webster *et al*. (2014b) also found that increased anxiety and depression were related to continued pain in NCCP, thus suggesting that patients with NCCP and psychological morbidity are at risk of persistent chest pain. Therefore, it may be useful to focus intervention efforts on patients with both NCCP and psychological morbidity, and the CSM may be helpful in developing such interventions. Further exploration of the nature of illness representations in patients with NCCP could therefore inform such interventions.

Previous interventions for patients with NCCP have been centred on cognitive behavioural therapy (CBT). Although CBT has been found to be effective (Kisely *et al*. 2012), it is typically intensive in nature, and thus difficult to deliver in an ED setting. There is therefore a need for brief psychological interventions. However, it has been suggested that such interventions may not be effective, due to a lack of acceptance of psychological factors, which is difficult to overcome in a brief intervention (Esler & Bock 2004). Webster *et al*. (2014b) found that a belief in psychological causes of chest pain was related to psychological morbidity in patients with NCCP, suggesting that patients may indeed be accepting of psychological causes of NCCP. This relationship therefore needs re‐examining in more detail, to determine whether brief psychological interventions may be appropriate for patients with NCCP and psychological morbidity.

To date, there have been no qualitative studies examining the illness representations of patients with NCCP, and only two qualitative studies that have examined patients' experiences of their condition and its treatment. Johnson *et al*. (2009) conducted interviews with patients with chest pain, to assess their experiences and reflections of the care that they received. Patients who received a noncardiac diagnosis experienced high levels of uncertainty and frustration, reflecting a lack of coherence (a dimension of the CSM), and wanted more information at point of diagnosis. However, while informative, the findings from this study were not linked to theory, which may limit the extent to which they improve our understanding of patients' experiences. Jerlock *et al*. (2005) used open‐ended, unstructured interviews to explore the daily life experience of patients with chronic NCCP. NCCP was found to have a strong negative impact (i.e. high perceived consequences) on patients' lives; for example, provoking fear (e.g. of myocardial infarction), restricting activity, impacting upon relationships and lifestyle, and causing stress and uncertainty.

There have been some qualitative investigations of illness representations in patients with other medically unexplained symptoms. Findings suggest that there may be a lack of clarity in illness representations in such conditions, particularly with regard to cause and identity (Green *et al*. 2004). It has been suggested that the lack of a clear label (identity) in medically unexplained symptoms leads to a lack of understanding across all other illness representation dimensions (Green *et al*. 2004).

### Aims

The present study used the CSM as a framework to provide an in‐depth exploration of the illness perceptions and the lived experience of patients with NCCP who continue to report chest pain and psychological distress. As NCCP patients with poor psychological outcomes are likely to be at risk of continued chest pain (Webster *et al*. 2014b), they are an important target for intervention.

## Methods

### Recruitment

The study received approval by a UK NHS Ethics Committee. Participants were recruited from those participating in a related quantitative study, which assessed relationships between illness representations and psychological and physical outcomes in NCCP (Webster *et al*. 2014b). For the quantitative study, participants were recruited from an ED if they were admitted with acute chest pain of suspected cardiac origin, were aged over 25, had no known coronary heart disease and had no other life threatening noncardiac pathology. The recruitment period ran from September 2010–July 2011. At the final follow‐up assessment (three months after diagnosis of NCCP), participants were asked whether they would be willing to be contacted regarding further research. Participants were considered for interview if they indicated that they were experiencing chest pain at least monthly at three‐month follow‐up, and scored ≥8 on either the anxiety or depression subscale of the Hospital Anxiety and Depression Scale (Zigmond & Snaith 1983) or >1 standard deviation below the sample mean on the SF‐12 assessment of QoL (Ware *et al*. 1996). These inclusion criteria were deliberately chosen to ensure that participants were experiencing continued chest pain as well as increased psychological distress and/or reduced QoL. These criteria were used to guide the purposeful sampling procedure described below. Duration of chest pain was not assessed, but this was discussed in the interviews.

### Participants and procedure

All participants who returned their final follow‐up questionnaire for the larger study were potentially eligible (*N *= 142). Of these participants, 60 agreed to be contacted regarding further research, of whom 18 were experiencing continued chest pain and increased levels of psychological morbidity. Potential participants were posted an information sheet regarding the study, and then contacted by telephone to enquire whether they would like to participate. Eight participants could not be contacted and three declined, providing a final sample size of seven. Interviews were arranged at a convenient time and location. Five participants opted to be interviewed on university premises, and two at home. Interviews were conducted by the first author. Participants received a copy of the information sheet in advance of the interview date, and informed consent was obtained prior to commencing interviews. The sample consisted of five females and two males with an age range from 40–76 (Median age = 49), which reflects the age and gender of NCCP patients in general (Webster *et al*. 2012, Smeijers *et al*. 2013).

### Data collection

A semi‐structured interview schedule was used to guide detailed interviews with the participants (see Table 1). To develop the interview schedule, appropriate questions were selected and adapted from qualitative studies that have investigated illness representations or experience of NCCP and other medically unexplained symptoms (Green *et al*. 2004, Jerlock *et al*. 2005). Participants were initially asked to give a biographical account of their experience of NCCP, describing their journey from when it started to the present day. This was done to ensure that questions and interviewing style would facilitate novel constructs (outside of the CSM) to emerge if present. After this, if the topics had not already arisen, questions were asked to assess specific illness representations. Interviews lasted between 20–75 minutes.

**Table 1:** Interview schedule questions

Topic	Questions
Probing for an account of individual's experience of NCCP	Try and think back to when you first experienced your chest pain. When, where and what did you first notice (probe for specific examples of early occurrences of the experience)?
	What happened when you went to see the doctors (probe for account of seeking help)?
	What has happened since then?
	Tell me about your last experience of chest pain.
Participants' current view or understanding of their chest pain (illness representations)	Do you feel you have a clear idea of what you are suffering from?
	What symptoms do you suffer from related to your chest pain?
	What do you think may have caused it?
	How do you feel about it?
	What do you think will happen with your chest pain in the future?
	What do you do when you experience your chest pain?

### Analysis

All interviews were recorded and transcribed verbatim. All transcripts were checked for accuracy against the recordings. Interviews were analysed using thematic analysis (Braun & Clarke 2006), which was deemed to be the most appropriate method, as it allows for both inductive and deductive themes to be identified/emerge, which therefore allows for identification of the CSM dimensions, as well as other themes related to patients' experiences of NCCP. The recruited sample was sufficient for the aim of generating an in‐depth understanding of participants' experiences, and is commensurate with other studies in this area (Green *et al*. 2004) and those that have used Thematic Analysis (e.g. Lo *et al*. 2008, Loke *et al*. 2012).

Analysis was undertaken by the first author with support of the second author. Initially, free coding was performed using illness representations as a template, but also coding for novel concepts. A very large amount of codes was produced at this point, to ensure novel constructs were not missed. These codes were then reviewed and collapsed together where possible, with some being integrated into the illness representation domains. Finally, these codes were reviewed thoroughly to determine whether there were any prominent novel themes outside the illness representation dimensions. Different aspects of the illness representation dimensions accounted for the majority of the data.

Transparency was maintained throughout the process, with a record being kept of all stages of data collation and analysis. An audit of analysis was performed, whereby the second author analysed two transcripts to assess comparability to the codes and themes derived by the first author, and to ensure that the process had been inclusive of all data and that the findings were warranted. Only minor discrepancies were identified, which were discussed and resolved easily. These methods have been widely recommended as approaches to ensuring that the analysis process has been rigorous (Mays & Pope 2000, Spencer & Ritchie 2012).

## Results

Six themes were identified in the transcripts that mapped onto CSM dimensions; namely, (1) identity, (2) cause, (3) timeline, (4), consequences (5) personal control and (6) treatment control. Emotional representations overlapped with the discussion of the consequences of the pain, and so are included within the discussion of this theme. An additional finding was that (a lack of) coherence, instead of emerging as a standalone theme (as dictated by the CSM), permeated throughout all dimensions (and is discussed as such).

### Identity

Participants generally did not have a name for their condition, or struggled to come up with one and, as such, there seemed to be a lack of clarity as to the identity of the condition.

Interviewer ‘Do you have a clear idea of what you're suffering from; do you have a name for it?’

P4 ‘No. Not that no, my other complaints yes, but not that no, I haven't got a clue what it is’

P7 ‘Do I have a name for it?…Erm…(sighs) No I can't think what I would call it. What would I call it?’

When participants did have a label for their condition, this was typically derived from what they had been told by clinicians and was usually described as being mechanical in nature.

P2 ‘I went to the physiotherapist and they said if it's not your heart, the only other thing it can be is muscular’

### Cause

Participants discussed holding a variety of beliefs about causes for their pain. The most prominent issue when discussing possible causes was a lack of understanding (coherence) about what was causing the pain, or how causal mechanisms might work (e.g. how stress might cause chest pain). Often participants had considered more than one cause, and some continued to hold a range of beliefs. Where participants had received an alternative (noncardiac) diagnosis (e.g. muscular pain), they often lacked understanding of what this meant. All participants initially considered a cardiac cause for their pain, often due to the suggestion of this by other people. These concerns were described as quickly subsiding for most participants; however, a small number still maintained a belief that their pain could be cardiac, and suffered anxiety and worry as a result of this. The majority of participants discussed physical causes for their pain (e.g. muscular, gastrointestinal); however, most were still uncertain about the mechanisms of these physical causes and the link between physical and psychological factors, again highlighting the lack of understanding within this dimension.

P6 ‘I don't really understand why [stress] causes chest pains’

P1 ‘Then [the paramedics] took me [to hospital]…I wasn't very worried by that time really, because he'd done the initial ECG here’

P7 ‘And they said everything was fine, I've been able to keep telling myself it's not my heart, it's ok…but then I've had this irrational fear’

Participants developed ideas about cause through diagnosis by a health professional and/or by making connections between events/feelings and the pain for themselves. Some participants, however, struggled to identify triggers to their pain.

P3 ‘I was trying to think, when it first happened, what I'd had to eat’

P2 ‘I don't know. I don't know what brings it on. It hurts now so nothing that I know of brings it on’

The majority of participants acknowledged psychological factors as playing a role in the cause and maintenance of their chest pain. Participants who had not identified a causal link between stress and pain still reported a number of sources of stress in their lives, suggesting that stress was seen as possibly playing a role. The extent to which participants understood the link between pain and psychological factors varied. Some had made explicit connections implicating stress/anxiety, and a small number had received a diagnosis that was psychological in nature. Some participants viewed stress solely as a causal factor in their NCCP, whereas others believed there to be a bidirectional relationship between stress and pain, whereby stress caused the pain, and the pain also worsened the stress. Some ruminated about their pain and anticipated the onset of pain episodes, which could serve to worsen or maintain their chest pain.

P6 ‘Now I can relate it to the stress levels whereas before I thought about it but I didn't really relate it […] but now I can definitely […] I had chest pain the other evening, but I had had quite a stressful day at work’

P7 ‘I worry about the pain so because I get all stressed, I think this is what's happening, the pain gets worse and then I worry more’

A large amount of the uncertainty with regard to cause was focussed around a lack of understand of the relationship between stress and pain. This, in turn, was related to worrying.

P6 ‘It still can be quite worrying […] It does come on when I'm stressed, but why would‐ the reasons as to why you would get a pain in your chest just because you're stressed’

### Timeline

The duration that participants had been suffering with chest pain varied greatly, from one month to a number of years.

P7 ‘just periodically really […] I couldn't tell you how often or when, but down the years really on and off’

Most participants expressed uncertainty about how long their pain might last, or had not considered it. This may reflect a potential lack of consideration or understanding of the course of the pain and how it might be controlled (either due to lack of knowledge or avoidance), which may impact on pain coping and management.

P6 ‘I don't know…maybe it'll just go away as quick as it came’

In addition, all participants described their pain as episodic; however, the nature, duration, and frequency of these episodes varied greatly, both within and between participants. This demonstrates the heterogeneous nature of NCCP.

P4 ‘I could go for a few weeks and not have one, and then I have a quite a few of them, and then it'll stop and might just get an odd one and then few weeks later have quite a lot’

P5 ‘Could be a couple of minutes, could be a few hours, could be all day’

### Consequences

Chest pain appeared to have at least some impact on all participants, varying from restriction of daily activities to fear of serious consequences (e.g. death, disability). The pain had a psychological impact on most of the participants, reflecting the emotional representations dimension of the CSM. This was largely related to worry or concern about the pain, particularly with regard to its potential cause; however, some also reported that their pain made them feel down or unhappy.

P5 ‘Am I going to be a cripple by time I'm sixty?…You know, that's the thing that I'm looking towards is the possibility of maybe in ten years or twenty years am I going to be a cripple?’

P2 ‘You get fed up of it, don't you, pain’

Some found that the pain impacted on the way they related to others by making them snappy, irritable or angry. This impacted on both relationships and working life. NCCP also impacted on working life in other ways, such as feeling overwhelmed by workloads or struggling physically to work. This clearly shows a wider impact of the pain beyond individual suffering.

P5 ‘I can get a bit snappy…with people…Noticeably at [work], sometimes with my girlfriend’

### Personal control

Participants had come up with variety of methods to control or cope with their pain (e.g. using a fan to cool down, physically manipulating the body, exercise). Many had used pain relief in an attempt to control their pain; however, this was either ineffective, or participants were reluctant to take it due to a dislike of taking too many tablets, or the effects it had on their mood.

P5 ‘I don't like taking painkillers, especially strong one's because they just zombify you, and I don't like being like that’

Some participants reported restricting activity (e.g. running, household chores) in an attempt to control their pain or to avoid an adverse cardiac event (e.g. heart attack, death), sometimes even despite health professional advice to the contrary. While this was effective for some in the short term, it may be unhelpful, as pain behaviours such as avoidance of activity can serve to maintain pain and disability (Vlaeyen & Crombez 1999).

P1 ‘It just gets worse and then I have to stop running…Because I think ‘if I carry on what's going to happen?’ (laughs) you don't know…I might fall down dead (laughs)’

P4 ‘While I've got this heaviness here, [I] don't want to move about too much… you think if it's muscular, you're making it worse aren't you, if you do’

Some participants reported using relaxation to cope with their chest pain, which was effective, although some did not have established methods for this.

P6 ‘I just sit down and try and relax’

Interviewer ‘Is there anything specific that you do to relax?’

P6 ‘Erm no probably just watch TV, or you know take myself off, by myself

Some saw managing the causes of pain as key to getting rid of their pain. This was mainly focussed on stress, with participants seeing a reduction in stress as key to reducing pain. This reflects the strong focus on psychological causes of chest pain.

P6 ‘Routine helps, you know we have quite a strict routine at home […] We've just had the holidays, so that's been a bit higgledy piggledy but now we're all back into [a routine], yeah that I think that helps’

### Treatment control

With regard to methods of controlling pain advised/prescribed by health professionals, participants had largely been prescribed only pain relief by GPs; however, one participant had received physiotherapy (self‐referred), and one had received psychological therapy or counselling. There was a lack of faith in treatment methods in reducing pain, most likely due to inefficacy or lack of treatment received. This may be because most treatment offered was physical (e.g. painkillers, physiotherapy). The one participant who had been prescribed psychological techniques (relaxation) found this very beneficial, although no other participants had had this opportunity (although some quoted using nonformalised methods of relaxation, see above).

P2 ‘[The physiotherapist] gave me some exercises…But it still comes on so whatever they gave me it hasn't made a difference’

P7 ‘The counsellor gave me this CD with relaxation techniques on…So that helps… If I can do that it will last for a shorter period of time’

## Discussion

This study sought to provide a detailed qualitative examination of the illness representations of patients with NCCP, and to understand the broader experience of living with NCCP. Participants’ experiences mapped reasonably well onto the dimensions of the CSM. Six themes were identified that covered patients’ perceptions of identity, cause, timeline, consequences, personal control and treatment control. Novel findings included the strong lack of coherence across all dimensions and the acceptance of psychological causes of pain.

Across all illness representation dimensions, there was an overwhelming lack of coherence (understanding). Lack of understanding of the NCCP diagnosis has been noted in previous qualitative studies (Johnson *et al*. 2009), and it may be the case that poorly formed illness representations lead to poorer psychological and physical outcomes. Therefore, the clarity of illness representations may be as important as their valence in NCCP patients. With regard to implications for treatment of patients, these results suggest that simple ‘rule out’ of cardiac causes is not sufficient, and that providing a clear explanation for the patient's NCCP may improve the clarity of illness representations and help to reduce uncertainty.

The majority of participants in the present study were aware of psychological causes of their chest pain. Previous studies have suggested that patients with NCCP may be reluctant to accept psychological causes for their pain, and that this could lead to resistance to psychological treatment (Esler & Bock 2004). The present findings suggest that this may not be the case, and that brief psychological interventions may therefore be acceptable to patients with NCCP. While participants were aware of a connection between psychological factors and pain, they often struggled to understand the potential mechanisms of this relationship; most likely due to lack of an explanation, suggesting that interventions aimed at providing an explanation of this relationship may be warranted. If staff could offer information about the mechanisms of other potential causes, this may be helpful to patients. Indeed, drawing connections between stress and pain was reported as helpful for some participants, and so this may be an effective aspect of any intervention for this group.

In addition to psychological causes, potential causes for chest pain in general were discussed extensively throughout the interviews. This is most likely because of the unexplained nature of NCCP, meaning that most participants had not been given a clear explanation of cause. Interestingly, cause has not been found to be a strong predictor of outcomes in other studies applying the CSM (Hagger & Orbell 2003). The prominent role of this dimension found here may be because NCCP does not have a predefined explanation, unlike other illnesses (Robbins & Kirmayer 1991). All participants had initially considered a cardiac cause for their pain, but for most, these concerns subsided quickly, which is contrary to previous findings that patients with NCCP generally continue to maintain fears about cardiac problems (Jerlock *et al*. 2005). This may be due to the acute setting of this study; in contrast, chronic patients who undergo outpatient cardiac investigations often have their cardiac concerns reinforced via repeated investigations, and even misdiagnosis or treatment (Mayou *et al*. 1999). Alternatively, this acceptance that there was not a cardiac cause may result from participants in the present study being open to accepting a role for psychological causation (e.g. stress, tension, panic). This differs to previous findings with patients with medically unexplained symptoms, showing that patients are overly worried about their symptoms, focus on potential physical causes, and often dismiss or are unaware of how psychological factors may impact upon their symptoms (Ring *et al*. 2005).

Participants generally did not have a label for their condition, nor did they seem to have a clear idea about the expected timeline of their pain. Consistent with previous findings (Jerlock *et al*. 2005), NCCP had a strong impact on various domains of participants’ lives (e.g. work, relationships), thus supporting the need to provide intervention for these patients. Interestingly, emotional representations were encompassed within the consequences theme. Methods of controlling pain were limited, with a general reluctance towards physical methods such as pain relief, suggesting that a more holistic approach to treatment of NCCP may be warranted.

The findings of this study hold implications for the use of the CSM in patients with NCCP (and potentially in patients with medically unexplained symptoms more generally). Interestingly, the current findings mirror the structure of the original version of the CSM, which outlined five core illness dimensions (i.e. identity, cause, timeline, consequences, control/cure). The additional components of coherence and emotional representations were subsequently added to the main measure that is used to assess illness perceptions – i.e. the Illness Perception Questionnaire–Revised (Moss‐Morris *et al*. 2002). The current findings suggest that the Illness Perception Questionnaire–Revised may need to be used and interpreted with caution when assessing the illness perceptions of patients with NCCP, taking into account the fact that patients may not have coherence or understanding within each dimension. Further research is also needed to examine whether emotional representations are a distinct construct separate from consequences, within the CSM.

## Relevance to clinical practice

Participants reported dissatisfaction with physical methods of pain relief, and a preference for psychological methods (e.g. relaxation). A more holistic approach to care might therefore be more appropriate, introducing other methods of coping with pain in the event that pain relief is ineffective or undesirable. Some participants attempted to minimise their pain by restricting their activity. This is common in chronic pain samples, and is often unhelpful and may worsen or maintain the pain through deconditioning (Vlaeyen & Crombez 1999). Reintroducing activity in a graded, paced manner may thus be beneficial and could be included in interventions for NCCP.

Given the lack of persistent concern for cardiac problems and acceptance of psychological factors found in the present study, brief psycho‐educational interventions that draw on the principles of CBT may be useful for this group. CBT interventions have been shown to be effective for reducing psychological distress and pain in patients with NCCP (Kisely *et al*. 2012); however, such interventions are lengthy (six to eight weeks), and therefore not amenable to acute settings. Brief interventions lend themselves to use within acute settings, where staff do not have the time to deal with patients’ concerns. CBT‐based self‐help is known to be widely effective for anxiety disorders (Webster *et al*. 2014a) and brief interventions have shown some efficacy in assisting patients in dealing with NCCP (Arnold *et al*. 2009). For a minority, who maintain concerns about cardiac causes, a brief psychological intervention may not be sufficient, and so a more intensive intervention, addressing causal beliefs, may be necessary for these patients. A model of stepped care could therefore be implemented for NCCP patients, whereby patients initially receive a less intensive therapy, incorporating CBT anxiety management‐based self‐help, giving patients methods to reduce their pain. Those who are nonresponsive could then be ‘stepped up’ to more intensive interventions, such as CBT, to tackle health anxiety (Mayou *et al*. 1999).

### Limitations

A number of study limitations need to be taken into account when interpreting the present findings. First, the research was grounded in one theoretical framework, which may have influenced the findings. However, the CSM was developed on the basis of qualitative work assessing patient experience, which identified five core dimensions, consistent with the present results (Leventhal *et al*. 1980), and the design of the present study actively sought to enable space for novel material to emerge. Second, the transferability of the findings is limited by the sample size and the context of the study being conducted within the UK health care system. Patients in the ED are examined and treated differently than in other health care settings, which may impact on their understanding of NCCP. This contextual issue needs to be considered when interpreting the findings. A third limitation is the heterogeneous nature of the sample, with regard to duration and nature of chest pain, and the diagnosis. While this may be considered a limitation, this might be unavoidable as NCCP is, by nature, a heterogeneous condition, with a variety of potential causes, including gastrointestinal, musculoskeletal and psychological. As such, it is therefore appropriate to group these patients by the general symptom of ‘noncardiac chest pain’, despite the potential heterogeneity.

## Conclusions

In conclusion, the experiences of NCCP in this study were largely consistent with the CSM, and provides an in‐depth picture of illness representations in people living with continued physical (i.e. NCCP) and psychological (e.g. anxiety) morbidity. The findings highlighted that (a lack of) coherence pervades into all illness representation dimensions. This lack of understanding was particularly pertinent within the cause dimension, such that participants were often accepting of psychological causes of their pain, but struggled to understand the mechanisms of this connection. Furthermore, patients also restricted their activity as a result of their chest pain.

## Disclosure

All authors were involved in the study design and manuscript preparation. Data analysis was conducted by RW and AT.

## Funding

The first author conducted this research as part of her PhD, which was funded by the Economic and Social Research Council and the Medical Research Council.

## Conflicts of interest

The author(s) declare that they have no conflict of interests.
